# Supplementary material for: Brucella effectors NyxA and NyxB target SENP3 to modulate the subcellular localisation of nucleolar proteins
Source: Nat Commun. 2023 Jan 6;14:102. doi: 10.1038/s41467-022-35763-8 (PMC9823007; doi:10.1038/s41467-022-35763-8)
Supplement: Supplementary file 1 — Supplementary information [file 41467_2022_35763_MOESM1_ESM.pdf]

Supplementary Table 1. Table of identified baits for NyxA.

Baits from Y2H for NyxA interaction partner	Number of hits
Homo sapiens SUMO1/sentrin/SMT3 specific peptidase 3 (SENP3)	5
Homo sapiens complement component 1, r subcomponent (C1R)	8
Homo sapiens canopy 4 homolog (zebrafish) (CNPY4)	2
TAF6 TAF6 RNA polymerase II, TATA box binding protein (TBP)-associated factor	1
CEBPZ CCAAT/enhancer binding protein (C/EBP), zeta [Homo sapiens]	3
ARL6IP4 ADP-ribosylation-like factor 6 interacting protein 4	3
Glutathione S-transferase kappa 1	1
Homo sapiens chromosome 11 genomic scaffold, alternate assembly HuRef SCAF_1103279188392	1
Homo sapiens chromosome 11 genomic scaffold, alternate assembly CHM1_1.0	
Homo sapiens chromosome 11 genomic contig, GRCh37.p10 Primary Assembly	
Heparan sulfate proteoglycan 2 (HSPG2)	1
Homo sapiens RNA binding motif, single stranded interacting protein 3 (RBMS3)	1
Homo sapiens alkaline phosphatase, intestinal (ALPI), mRNA	1
Homo sapiens alkaline phosphatase, placental-like 2 (ALPPL2), mRNA	
Homo sapiens alkaline phosphatase, placental (ALPP), mRNA	

Supplementary Table 2. Data collection and refinement statistics

Statistics for the highest-resolution shell are shown in parentheses.

	**Se-Met**	**Native**
Wavelength (Å)	0.97930	0. 97242
Resolution range (Å)	48.94 - 3.7 (3.9 - 3.7)	48.69- 2.5 (2.64 - 2.5)
Space group	P 62 2 2	P 61 2 2
Cell parameters a, b, c (Å)	77.60 77.60 195.08	133.91 133.91 389.51
Total reflections	101555 (15006)	1420875 (194308)
Unique reflections	4203 (590)	72440 (10381)
Multiplicity	24.2 (25.4)	19.6 (18.7)
Completeness (%)	100.0 (98.6)	100.00 (100.00)
Mean I/sigma(I)	17.4 (4.4)	12.5 (2.9)
Wilson B-factor	120.0	35.9
R-merge	0.133 (0.805)	0.164 (1.035)
R-meas	0.138	0.173
CC1/2	0.999 (0.972)	0.996 (0.958)
**Refinement**		
R-work		0.21 (0.27)
R-free		0.24 (0.33)
Macromolecules		11046
Ligands		0
Water		519
Protein residues		1405
RMS (bonds)		0.013
RMS (angles)		1.33
Ramachandran favored (%)		98.6
Ramachandran allowed (%)		1.4
Ramachandran outliers (%)		0
Clashscore		3.42
Average B-factor		51.84
macromolecules		52.01
solvent		48.16

Supplementary Table 3. Small-angle X-ray scattering data collection and analysis

	Range	Rg (nm)	sRg limits	I0 (cm^{-1})	Porod Volume	Dmax (nm)
**NyxB**						
Guinier	34-81	2,9 +/- 0,037	0,60/1,24	3,21 +/- 0,02		
Gnom	20-380	3		3,0	37,8	10,2
**NyxA**						
Guinier	40-91	2,720 +/- 0,024	0,64/1,3	4,06 +/- 0,02		
Gnom	28-590	2,82		2,83	39,6	9,6

Supplementary Table 4. Statistical analysis of Figure 5B.

Tukey's multiple comparisons test	Summary	P Value
Negative vs. wild-type	****	<0,0001
Negative vs. ΔnyxAnyxB	**	0,0081
wild-type vs. ΔnyxAnyxB	**	0,0036
wild-type vs. Δ*nyxA*	*	0,0247
wild-type vs. Δ*nyxA:Tn7-nyxA*	ns	0,9984
wild-type vs. Δ*nyxA*:Tn7-*nyxA*^{MAG}	*	0,0147
wild-type vs. Δ*nyxB*	*	0,0353
wild-type vs. Δ*nyxB:*Tn7-*nyxB*	ns	0,9862
wild-type vs. Δ*nyxB:*Tn7-*nyxB*^{MAG}	*	0,0353
Δ*nyxA* vs. Δ*nyxA:*Tn7-*nyxA*	**	0,006
Δ*nyxA* vs. Δ*nyxA*:Tn7-*nyxA*^{MAG}	ns	>0,9999
Δ*nyxA:*Tn7-*nyxA* vs. Δ*nyxA*:Tn7-*nyxA*^{MAG}	**	0,0035
Δ*nyxB* vs. Δ*nyxB:*Tn7-*nyxB*	**	0,0051
Δ*nyxB* vs. Δ*nyxB:*Tn7-*nyxB*^{MAG}	ns	>0,9999
Δ*nyxB*:Tn7-*nyxB* vs. Δ*nyxB:*Tn7-*nyxB*^{MAG}	**	0,0051

Supplementary Table 5. *Brucella* strains used in this study.

Strain name	Description	Genetic features	Resistance
Brucella abortus 2308	Wild-type (obtained from X. de Bolle)	-	Nalidixic acid natural resistance
B. abortus DSRed		pTn7-DSRed	Kanamycin
Δ*virB9*	Deletion of *virB9*		
Wt p*bla:nyxA*	TEM1 translocation	pFlagTEM1-NyxA	Chloramphenicol
Δ*virB9* p*bla:nyxA*	TEM1 translocation	pFlagTEM1-NyxA	Chloramphenicol
Wt p*bla:nyxB*	TEM1 translocation	pFlagTEM1-NyxB	Chloramphenicol
Δ*virB9* p*bla:nyxB*	TEM1 translocation	pFlagTEM1-NyxB	Chloramphenicol
Wt p*bla:BAB1_0466*	TEM1 translocation	pFlagTEM1-BAB1_0466	Chloramphenicol
Δ*nyxA*	Deletion of *BAB1_0296 (nyxA)*		
Δ*nyxB*	Deletion of *BAB1_1101 (nyxB)*		
Δ*nyxAnyxB*	Deletion of both		
Δ*nyxA:Tn7-nyxA*	Complemented strain	pTn7-*nyxA*	Kanamycin
Δ*nyxA:Tn7-nyxA*^{MAG}	Complemented strain with mutated acidic groove (MAG)	pTn7-*nyxA*^{MAG}	Kanamycin
Δ*nyxA:Tn7-nyxB*	Complemented strain	pTn7-*nyxB*	Kanamycin
Δ*nyxB:Tn7-nyxB*^{MAG}	Complemented strain with mutated acidic groove (MAG)	pTn7-*nyxB*^{MAG}	Kanamycin
Δ*nyxA*p4HA-NyxA	Effector imaging	pBBR1MCS-4-4HA-*nyxA*	Ampicilin
Δ*nyxA*p3Flag-NyxA	Effector imaging	pBBR1MCS-4-3Flag-*nyxA*	Ampicilin
Δ*nyxB*p4HA-NyxB	Effector imaging	pBBR1MCS4-4HA-*nyxB*	Ampicilin

Supplementary Table 6. Primers and siRNA used in this study.

		**Sequence 5'-3'**
pNPTS138-*nyxA*	Fw1	GATAATGACGCTAGCTTTCA
	Rev1	GGCCACCCGACAAGCAGTTTGGAGGTTTACCTTTTGTTGA
	Fw2	TCAACAAAAGGTAAACCTCCAAACTGCTTGTCGGGTGGCC
	Rev2	GGGTGTCCTTGAAACATCCA
pNPTS138-*nyxB*	Fw1	TTGGATCAATCCGGCGTGTG
	Rev1	GGGCTTCAACTTCTTTAACCGGCTATTCTTCCTGTCAATT
	Fw2	AATTGACAGGAAGAATAGCCGGTTAAAGAAGTTGAAGCCC
	Rev2	CGTAAACGCTTCGGCAGGGA
pFlagTEM1-*nyxA*	fw	TAAGCATTGGTCTAGAATGAACGCTCACACAAACATAA
	rev	ACTGCAGTTATCTAGATCAAAGCTCCAAGCATCTAATT
pFlagTEM1-*nyxB*	fw	GAGATAGGTGCCTCACTGATTAAGCATTGGTCTAGAATGAACACGCA AGCAACAATA
	rev	GTGTGCTGGAATTCGCCCTTACTGCAGTTATCTAGATCAAGGCATCT CGATAAG
pFlagTEM1-BAB1_0466	fw	TAAGCATTGGTCTAGAATGAAAATGTGGACCCTTGC
	rev	ACTGCAGTTATCTAGATCACTGTTCTACGCAGCTTA
pTn7-*nyxA*	fw	AAAAAAGAGCTCTAAGTGTCTGCCATAGCCGACG
	rev	AAAAAGGATCCTCAAAGCTCCAAGCATCTAATTTC
pTn7-*nyxB*	fw	AAAAAAGAGCTCTTGGATCAATCCGGCGTGTGC
	rev	AAAAAGGATCCTCAAGGCATCTCGATAAGGC
pMMB 207c-*nyxA*	fw	AAAAAAGGTACCAACGCTCACACAAACATAAGTGG
pMMB 207c-*nyxA*	rev	AAAAAAAAGCTTTCAAAGCTCCAAGCATCTAATTTC
pMMB 207c-*nyxB*	fw	AAAAAAGGTACCAACACGCAAGCAACAATAGATACAGC
pMMB 207c-*nyxB*	rev	AAAAAAAAGCTTTCAAGGCATCTCGATAAGGC
pBBRMCS4 4HA-*nyxA*	fw	AAAAAAGAGCTCAAGGAGATATACATATGTACC
	rev	AAAAAAACTAGTTCAAAGCTCCAAGCATCTAATTTC
pBBRMCS4 3Flag-*nyxA*	fw	TATTCCCGGGGGATCCATGGGTAAGCCTATCCCTAACCCTCTCCTCG GTCTCGATT CTACGAACGCTCACACAAACATAAG
	rev	TATAGGGCGAATTGGAGCTCAAGCTCCAAGCATCTAATTT
pBBRMCS4 4HA-*nyxB*	fw	AAAAAAGAGCTCAAGGAGATATACATATGTACC
	rev	AAAAAAACTAGTTCAAGGCATCTCGATAAGGC
pDONOR-*nyxA*	fw	GGGGACAAGTTTGTACAAAAAAGCAGGCTTCAACGCTCACA CAAACATAAG
	rev	GGGGACCACTTTGTACAAGAAAGCTGGGTCCTAAAGCTCCAAGCATC TAATTTC
pDONOR-*nyxB*	fw	GGGGACAAGTTTGTACAAAAAAGCAGGCTTCAACACGCAAGCAACAA TAGATA
	rev	GGGGACCACTTTGTACAAGAAAGCTGGGTCCTAAGGCATCTCGATAA GGCGGATT
pcDNA3.1-4HA-*nyxA*	Fw	GCGTTTAAACTTAAGCTTGGTACCGAGCTCGGATCCAAGGAGATATA CATATGTACC
	Rev	CGAGCGGCCGCCACTGTGCTGGATATCTGCAGAATTCTCAAAGCTC CAAGCATCTAATTTC
pcDNA3.1-4HA-*nyxB*	Fw	GCGTTTAAACTTAAGCTTGGTACCGAGCTCGGATCCAAGGAGATATA CATATGTACC
	rev	CGAGCGGCCGCCACTGTGCTGGATATCTGCAGAATTCTCAAGGCAT CTCGATAAGGC
pET151His-*nyxA*	fw	CACCATGAACGCTCACACAAAC
	rev	TCAAAGCTCCAAGCATCT

pET151His-*nyxB*	fw	CACCATGAACACGCAAGCAAC	
	rev	CATTATGCTCCCCTGTTGT	
NyxA Y62R	fw	CGATTGGCGACCTGCCGCCTATGATG	
	rev	CGGCAGGTCGCCAATCGTAGCAGTCGAAG	
NyxA D76R	fw	CCATGAAACGACGGGAACTGATCCAATACG	
	rev	TTCCCGTCGTTTCATGGCGTTGCCTTC	
NyxA E78R	fw	CGACGGAGACTGATCCAATACGAAGAGTGGTG	
	rev	GGATCAGTCTCCGTCGTTTCATGGCG	
SENP3 $_{7\text{-}159}$	Fw	CACCATGGCCGGCACCGGTAGCTGGGGTCCGGAACC	
	rev	TTTGGATCCTTATTTGCTATACAGCAGCATACGAAATGC	
NyxB D80R	fw	CCATGAAACGACGGGAACTGATCCAATATGAAG	
	rev	GTTCCCGTCGTTTCATGGCGTTGCCTTC	
NyxB E82R	fw	AACGACGGAGACTGATCCAATATGAAGATTGGTGC	
	rev	GGATCAGTCTCCGTCGTTTCATGGCGT	
NyxB Y66R	fw	CGACTGGCGACCCGCCGCCTATGACGAC	
	rev	GCGGGTCGCCAGTCGTAGGTGTCGAAGAAGAAGC	
siRNA			
siSENP3(a)	Life Technologies	AAACUCCGUACCAAGGGUUAU	
siSENP3(b)	Dharmacon-Horizon Discovery	ON-TARGET plus Human SENP3 (26168) siRNA – SMARTpool L-006034-00-0005	ACGAAUUCCUUCAAACGUA<br>GCACUGAUGAGGUAGUAGA<br>GAUAAACUCCGUACCAAGG<br>CAAGUCAGGUGGAGGGUUU
siPIAS3	Dharmacon-Horizon Discovery	ON-TARGET plus Human PIAS3 (10401) siRNA – SMARTpool L-004164-00-0005	GAGCCGACAUCCAAGGUUU<br>UAAGAAGAAGGUCGAAGUU<br>GGAAGCGCACUUUACCUUU<br>GACAGAGAGUCAGCACUAU
siBeclin	Dharmacon-Horizon Discovery	ON-TARGET plus Human BECN1 (8678) siRNA – SMARTpool L-010552-00-0005	GAUACCGACUUGUUCUUA<br>GGAACUCACAGCUCCAUUA<br>CUAAGGAGCUGCCGUUAUA<br>GAGAGGAGCCAUUUAUUGA
siNVL	Dharmacon-Horizon Discovery	CCAGGGAAAGAAUACUUCAAACAUU	
siRPL5	Dharmacon-Horizon Discovery	CUACCACUGGCAAUAAAG	
siControl	Dharmacon-Horizon Discovery	ON-TARGET plus NON-targeting Pool D-001810-10-05	UGGUUUACAUGUCGACUAA<br>UGGUUUACAUGUUGUGUGA<br>UGGUUUACAUGUUUUCUGA<br>UGGUUUACAUGUUUUCCUA

Supplementary Table 7. Antibodies used in this study.

Antibody	Species	Source	Reference	Dilution	Fixation
SENP3	Rabbit	Cell Signalling	5591	1/400	PFA
NVL	Rabbit	Produced by M. Nagahama lab		1/50	Antigenfix
NVL	Rabbit	Proteintech	16970-1-AP Batch 00008428	1/25	Antigenfix
RPL5	Rabbit	Produced by M. Nagahama lab		1/100	Antigenfix
PES1	Rabbit	ATLAS antibodies	HPA040210	1/100	Antigenfix
NPM1	Rabbit	Abcam	Ab37659	1/400	PFA
Nucleolin	Mouse	Invitrogen	39-6400	1/100	Antigenfix
HA	Rat	Roche	#11867423001 clone 3F10	1/100	PFA/Antigenfix
Flag	Mouse	Sigma	F1804 (clone M2)	1/2000	Antigenfix/methanol
Myc	Mouse	DSHB	Clone E10	1/1000	PFA/Antigenfix
NUFIP1	Rabbit	Proteintech	12515-1-AP	1/50	Antigenfix
LAMP1	Mouse	DSHB	Clone H4A3	1/200	PFA/Antigenfix

FK2	Mouse	Enzo	BML-PW8810	1/1000	Antigenfix
g3bp (H10)	Mouse	Santa Cruz	sc-365338	1/25	PFA
eiF3n (c-5)	Mouse	Santa Cruz	sc-137214	1/50	PFA
Xrnl (C-1)	Mouse	Santa Cruz	sc-165985	1/25	PFA
DDX20 (Gemin3-12H12)	Mouse	Santa Cruz	sc-57007	1/50	PFA
LC3	Mouse	Nanotools	0231-100/LC3-5F10	1/1000	Methanol
Western blot					
Actin	Mouse	Sigma	A4700	1/1000	
His	Mouse	Sigma	H1029	1/3000	
V5	Mouse	Invitrogen	R960-25	1/1000	
SENP3	Rabbit	Cell Signalling	5591	1/400	
NPM1	Rabbit	Abcam	Ab37659	1/400	
Histone 3	Rabbit	Abcam	Ab8895	1/500	
NVL	Rabbit	Proteintech	16970-1-AP Batch 00008428	1/25	
PIAS3(D5F9) XP	Rabbit	Cell Signalling	9042	1/1000	
Beclin	Rabbit	Abcam	Ab62557	1/1000	
Secondary antibodies					
Donkey anti-mouse AlexaFluor488/555/647		Invitrogen	A-21202/A-31570/A-31571	1/1000	
Donkey anti-rabbit AlexaFluor488/555/647		Invitrogen	A-21206/A-31572/A-31573	1/1000	
Donkey anti-rat AlexaFluor488/555/647		Invitrogen	A-21208/A-21434/A-21472	1/1000	
Anti-rabbit HRP linked antibody		Cell signaling	7074S	1/5000	
Anti-Mouse-HRP		Cytiva	NA931	1/5000	

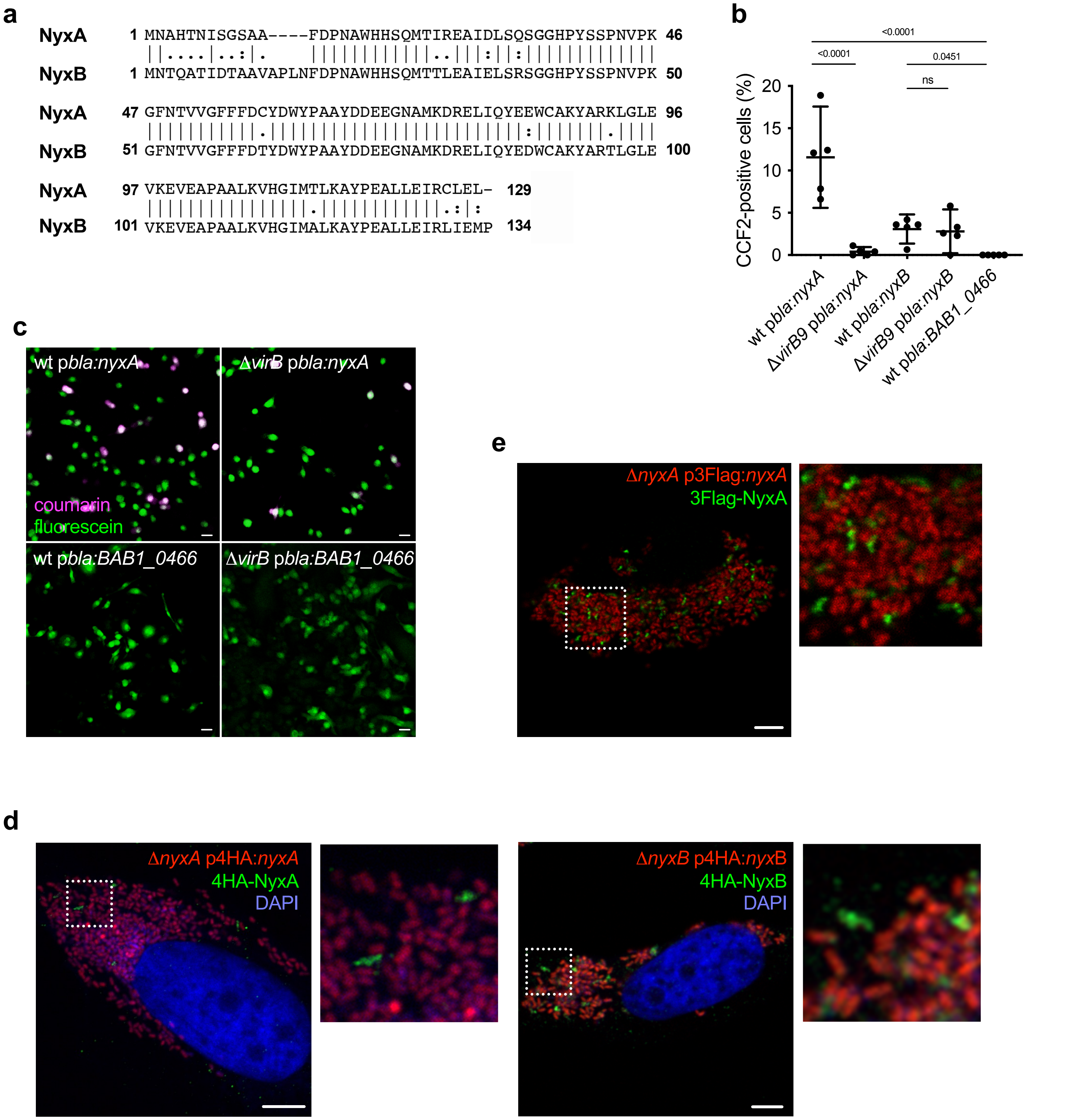

Supplementary Figure 1. NyxA and NyxB are translocated into host cells during infection, accumulating in punctate or filament-like structures. (a) Amino acid sequence alignment of NyxA and NyxB obtained with ClustalW, matrix EBLOSUM62, showing 82.1% identity, 86.6% similarity and 3.7% gaps. **(b)** RAW macrophage-like cells were infected for 4h with *B. abortus* wild-type or *virB9* expressing TEM1 (encoded by the *bla* gene) fused with NyxA, NyxB or BAB1_0466. The percentage of cells with coumarin emission, which is indicative of translocation, was quantified after incubation with the CCF2-AM substrate. Data represent means ± 95% confidence intervals from 5 independent experiments, with more than 300 cells counted for each condition. A Kruskal-Wallis test with Dunn's correction was used for comparisons. **(c)** Representative images for *B. abortus* wild-type or Δ*virB9* carrying p*bla:nyxA* or p*bla:nyxB* to exemplify the presence of effector translocation visible in coumarin-positive cells (violet) or absence of translocation. **(d)** Representative images to illustrate the filament-like structures visible with both 4HA-tagged NyxA (left) or NyxB (right). HeLa cells were infected for 48h with Δ*nyxA* or Δ*nyxB* strains expressing DSRed and carrying p4HA*:nyxA* or p4HA*:nyxB*. The cell nucleus is visible with DAPI (blue). **(e)** Representative confocal microscopy images showing accumulation of 3Flag-NyxA (green) punctate structures in HeLa cells infected for 48h with Δ*nyxA* expressing DSRed and 3Flag-NyxA. All scale bars correspond to 5 μm.

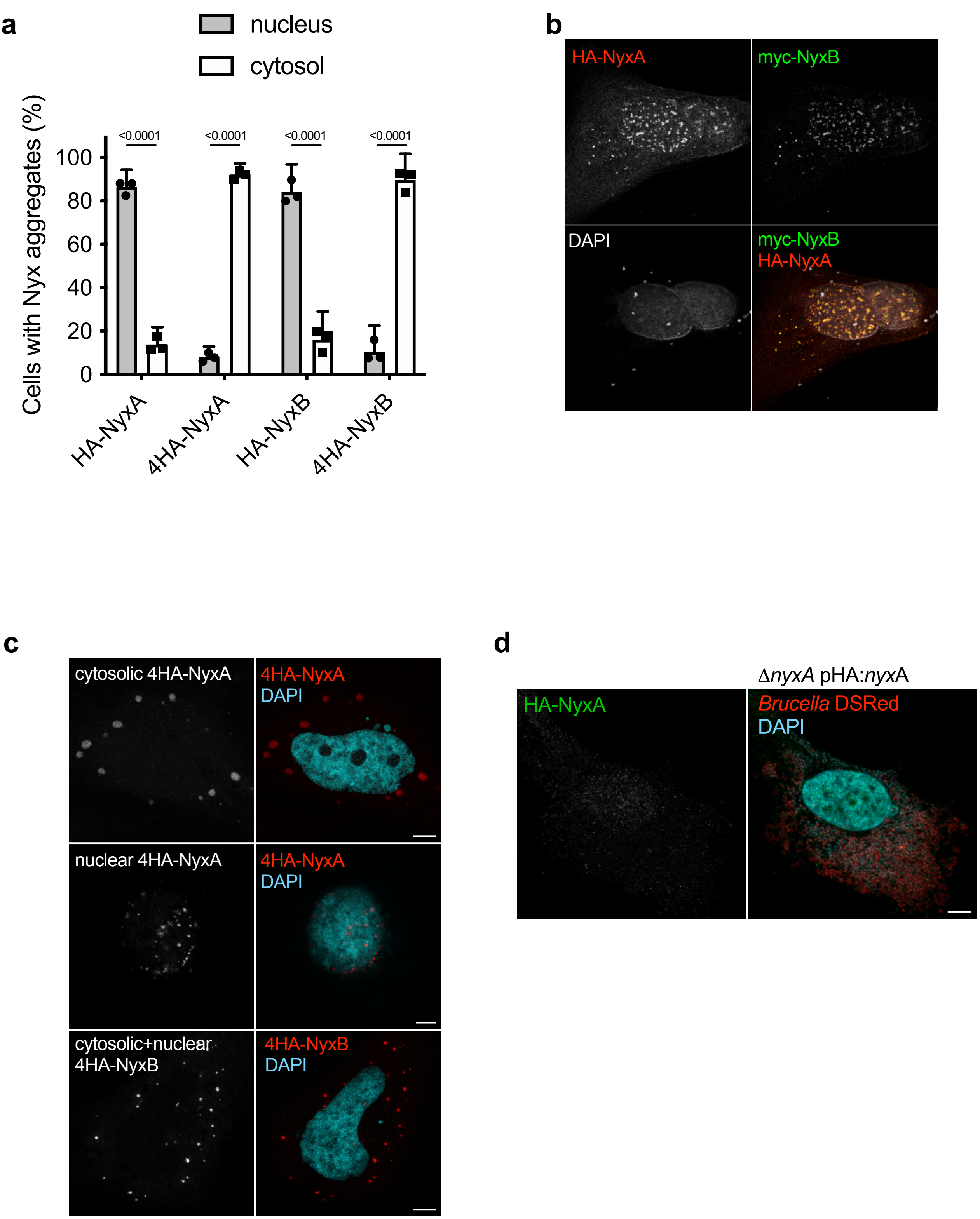

Supplementary Figure 2. NyxA and NyxB target the same cellular compartments. (a) Quantification of the percentage of cells with the majority of tagged effector accumulating in cytoplasmic or nuclear structures. Data correspond to means 95% confidence intervals from 3 independent experiments. A two-way ANOVA was used with Bonferoni correction for selected comparisons. **(b)** Representative confocal microscopy images of HA-NyxA (red) and Myc-NyxB (green) ectopically expressed in HeLa cells showing cytosolic and nuclear co-localization. **(c)** Representative confocal microscopy images of 4HA-tagged NyxA or NyxB (red) ectopically expressed in HeLa cells showing cytosolic or nuclear localizations. **(d)** Absence of detection of translocated HA-NyxA during infection. All scale bars are 5 μm.

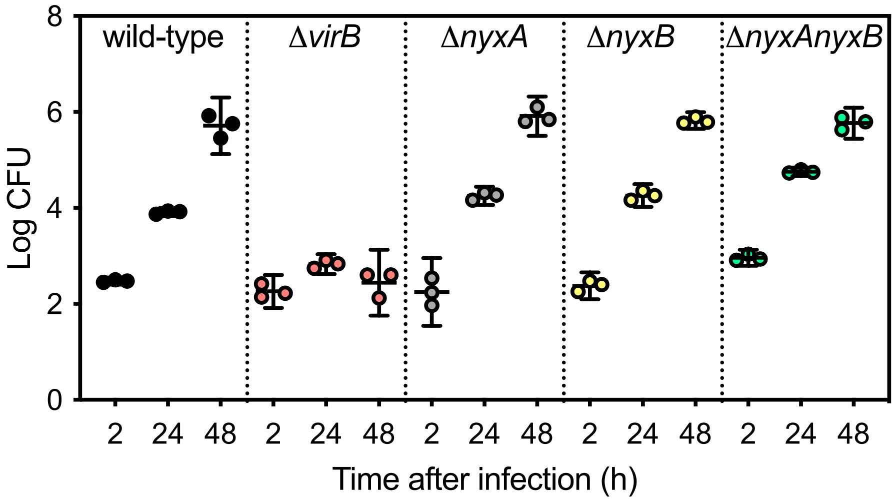

Supplementary Figure 3. Deletion of NyxA and NyxB does not impact the intracellular multiplication of *B. abortus*. Enumeration of bacterial colony forming units (CFU) of wild-type *B. abortus*, *virB9*, *nyxA*, *nyxB* or *nyxAnyxB* following 2, 24 or 48h of infection of HeLa cells. Data correspond to means ± 95% confidence intervals from 3 independent experiments.

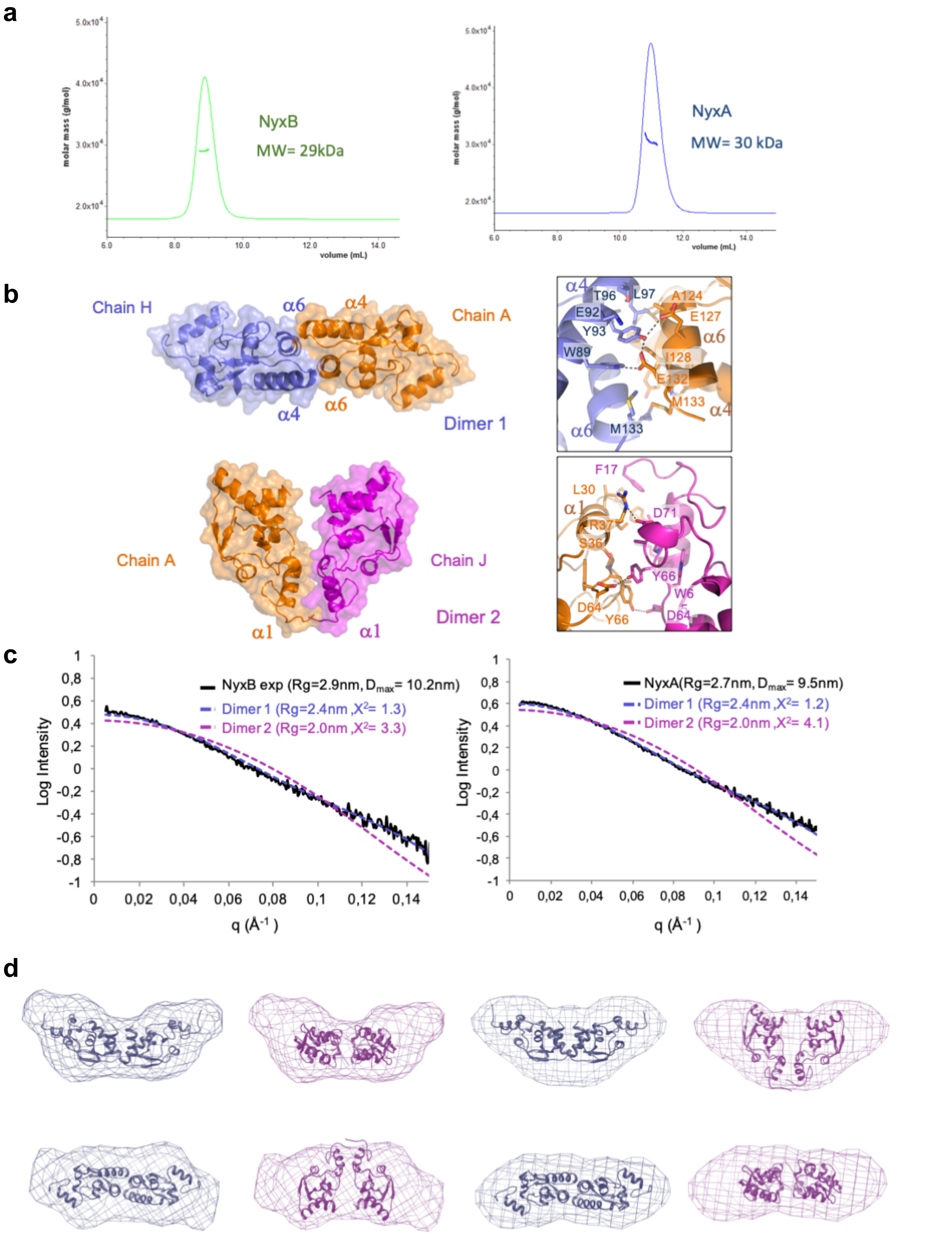

Supplementary Figure 4. NyxA and NyxB dimer formation. (a) Chromatograms ($A_{280,}$ plain) and mass measurements (dots) by Multi-Angle Light Scattering of NyxB (left, green) and NyxA (right, blue). The average molecular weight determined is indicated. **(b)** Surface representation of dimer A (chains A and H) and dimer 2 (chains A and J) with a detailed view of each interface side chains involved represented as ball-and-sticks. **(c)** Comparison of the theoretical small-angle X-ray scattering profiles of NyxB dimers with experimental data (black curves) obtained for NyxB (left) and NyxA (right). Fitting values (c2) obtained using FoXS server are indicated. **(D)** Fitting of NyxB dimers into ab initio SAXS envelopes obtained with GASBOR using NyxB data (left) and NyxA (right) showing that dimer 1 fits much better each of the experimental curves.

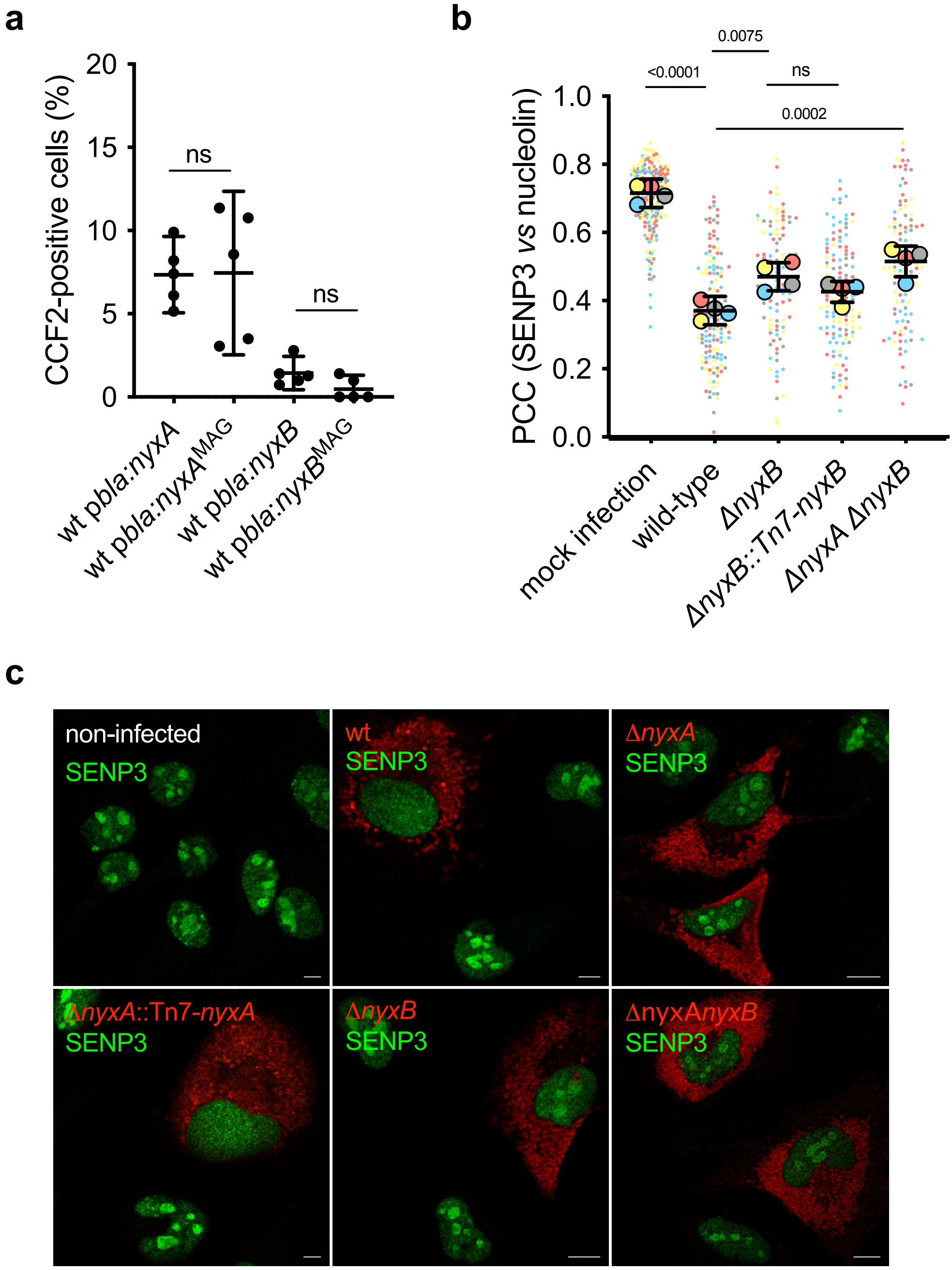

Supplementary Figure 5. The *Brucella* Nyx effectors directly reduce the SENP3 nucleolar localisation in host cells. (a) RAW macrophage-like cells were infected for 24h with *B. abortus* wild-type expressing TEM1 (encoded by the *bla* gene) fused with NyxA, NyxA^{MAG}, NyxB or NyxB^{MAG}. The percentage of cells with coumarin emission, which is indicative of translocation, was quantified after incubation with the CCF2-AM substrate. Data represent means ± 95% confidence intervals from 5 independent experiments. A Kruskal-Wallis test with Dunn's correction was used for comparisons. **(b)** Quantification of the Pearson's coefficient of SENP3 versus nucleolin in HeLa cells infected for 48h with either *B. abortus* wild-type or *nyxB*, its complemented strain *nyxB*::Tn7-*nyxB* or double deletion mutant *nyxAnyxB*. Data are represented as means 95% confidence intervals from 4 independent experiments. Each experiment is colour coded and all events counted are shown. Data were analysed using one-way ANOVA by including all comparisons with Tukey's correction. Not all comparisons are shown. The p values are indicated and non-significant (ns) correspond to p>0.05. **(c)** Representative confocal microscopy images of HeLa cells infected with the different strains expressing DSRed and labelled for SENP3 (green), in comparison to mock-infected control cells (non-infected first panel). All scale bars correspond to 5 μm.

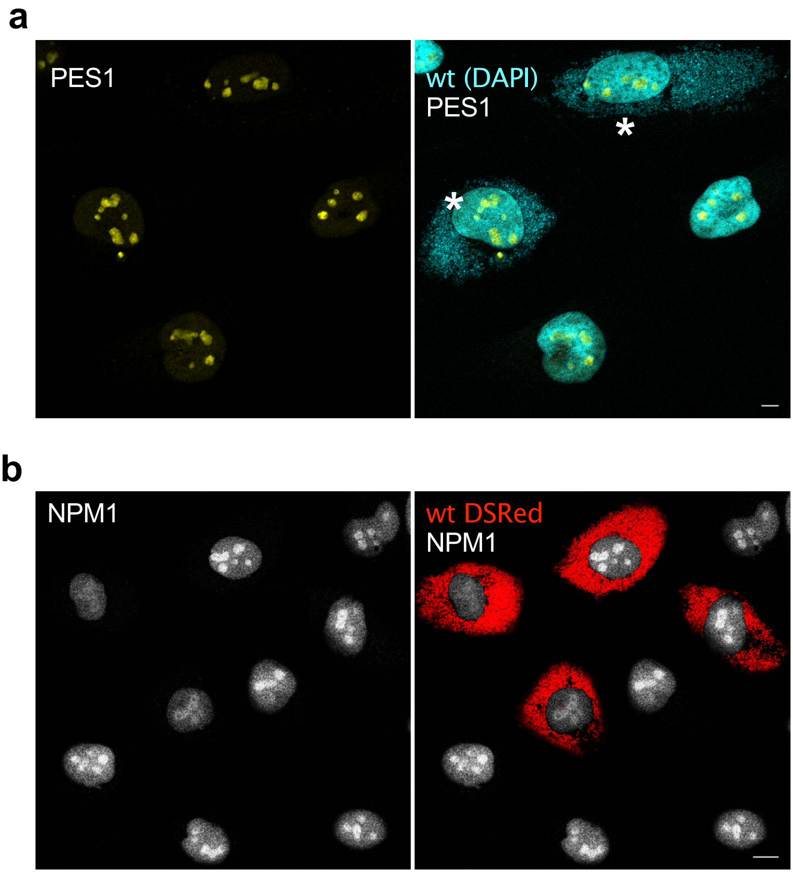

Supplementary Figure 6. *B. abortus* effect on PES1 and NPM1 nucleolar localization. (a) Representative confocal images of HeLa cells infected for 48h with wild-type *B. abortus* and labelled for DNA to visualise bacteria and cell nuclei (cyan) and PES1 (yellow). Infected cells are indicated with an asterisk. (b) HeLa cells were infected for 48h with DSRed-expressing wild-type *B. abortus* and labelled for NPM1 (white). All scale bars correspond to 5 μm.

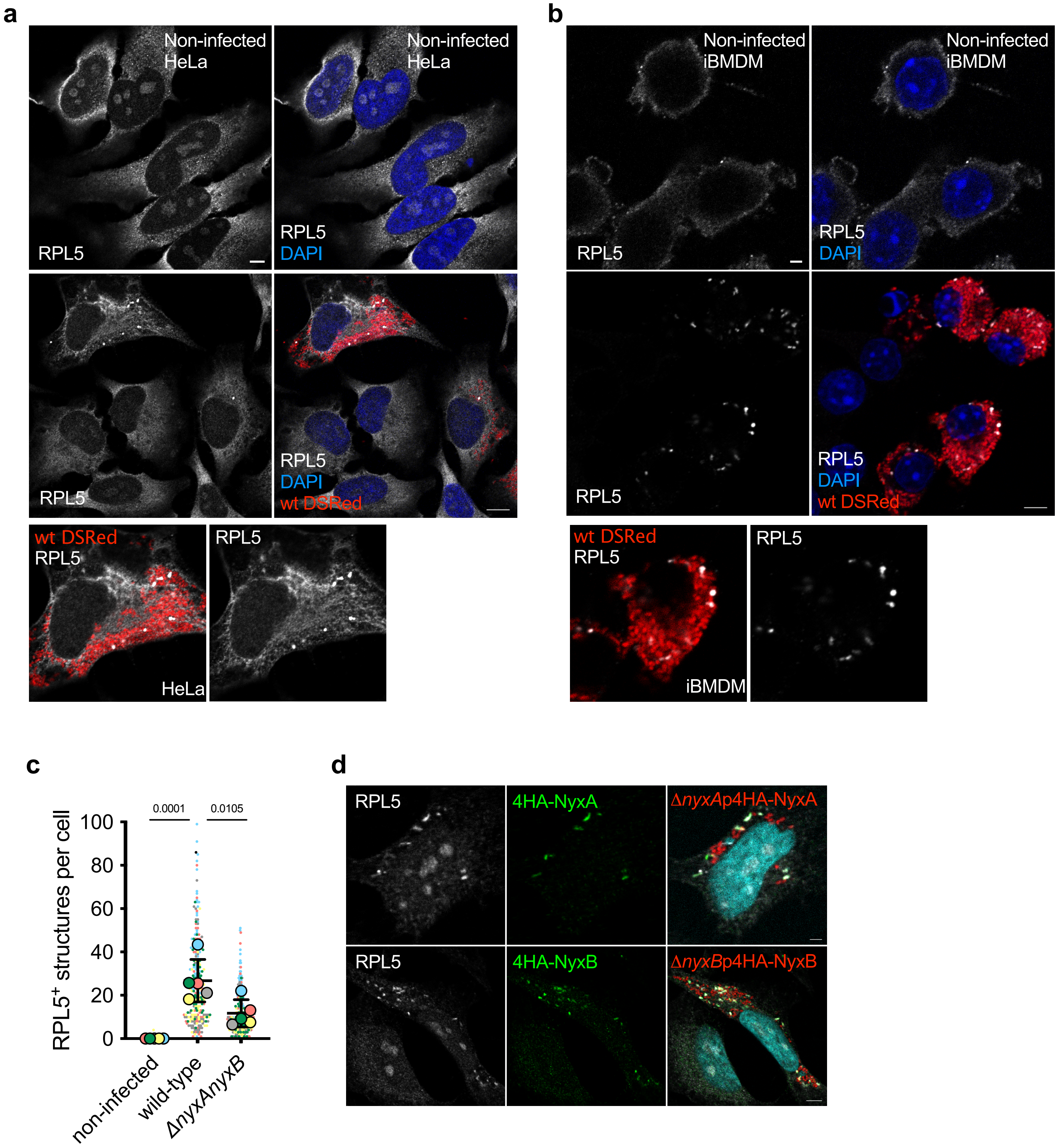

Supplementary Figure 7. *B. abortus* induces cytoplasmic accumulation of RPL5. Representative confocal microscopy images of **(a)** HeLa cells and **(b)** iBMDM infected with wild-type DSRed-expressing *B. abortus* for 48h and labelled for RPL5 (white) and DAPI (blue). Zoomed cells are included on the right. Non-infected cells are included as controls. **(c)** Quantification of the number of RPL5-positive cytoplasmic structures in HeLa cells infected for 48h with wild-type *B. abortus* or a mutant strain lacking NyxA/B. Mock-infected cells are included as a control. Data are represented as means 95% confidence intervals from 5 independent experiments. Each experiment is colour coded and all events counted are shown. Data were analysed using one-way ANOVA by including all comparisons with Tukey's correction. **(d)** Representative confocal microscopy images of HeLa cells infected for 48h with either *nyxA* expressing DSRed and 4HA-NyxA (top) or *nyxB* expressing DSRed and 4HA-NyxB and labelled for RPL5 (white) and DAPI (cyan). All scale bars correspond to 5 µm.

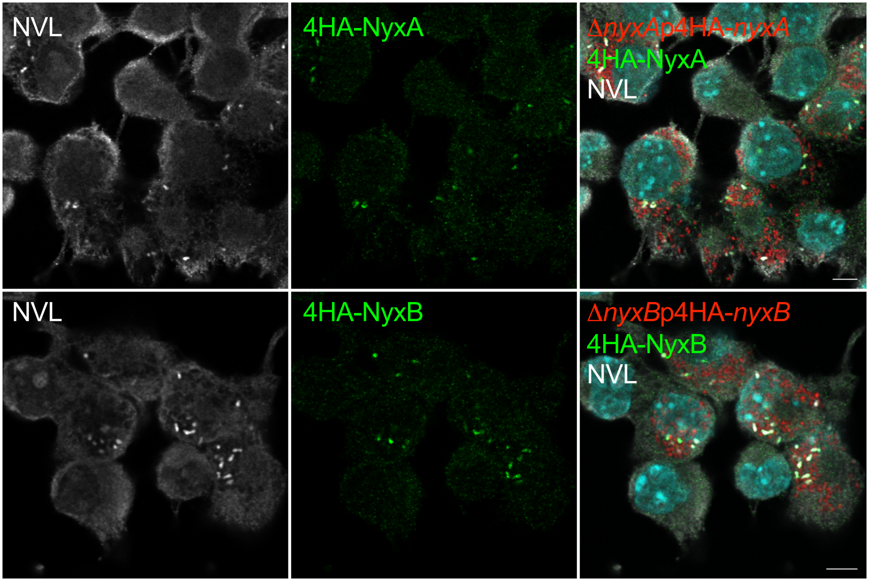

Supplementary Figure 8. *B. abortus* induces cytoplasmic accumulation of NVL in iBMDM that colocalises with translocated 4HA-tagged NyxA and NyxB. iBMDM were infected with either *nyxA* expressing DSRed and 4HA-NyxA (top) or *nyxB* expressing DSRed and 4HA-NyxB for 48h and labelled for NVL (white) and DAPI (cyan). All scale bars correspond to 5μm.

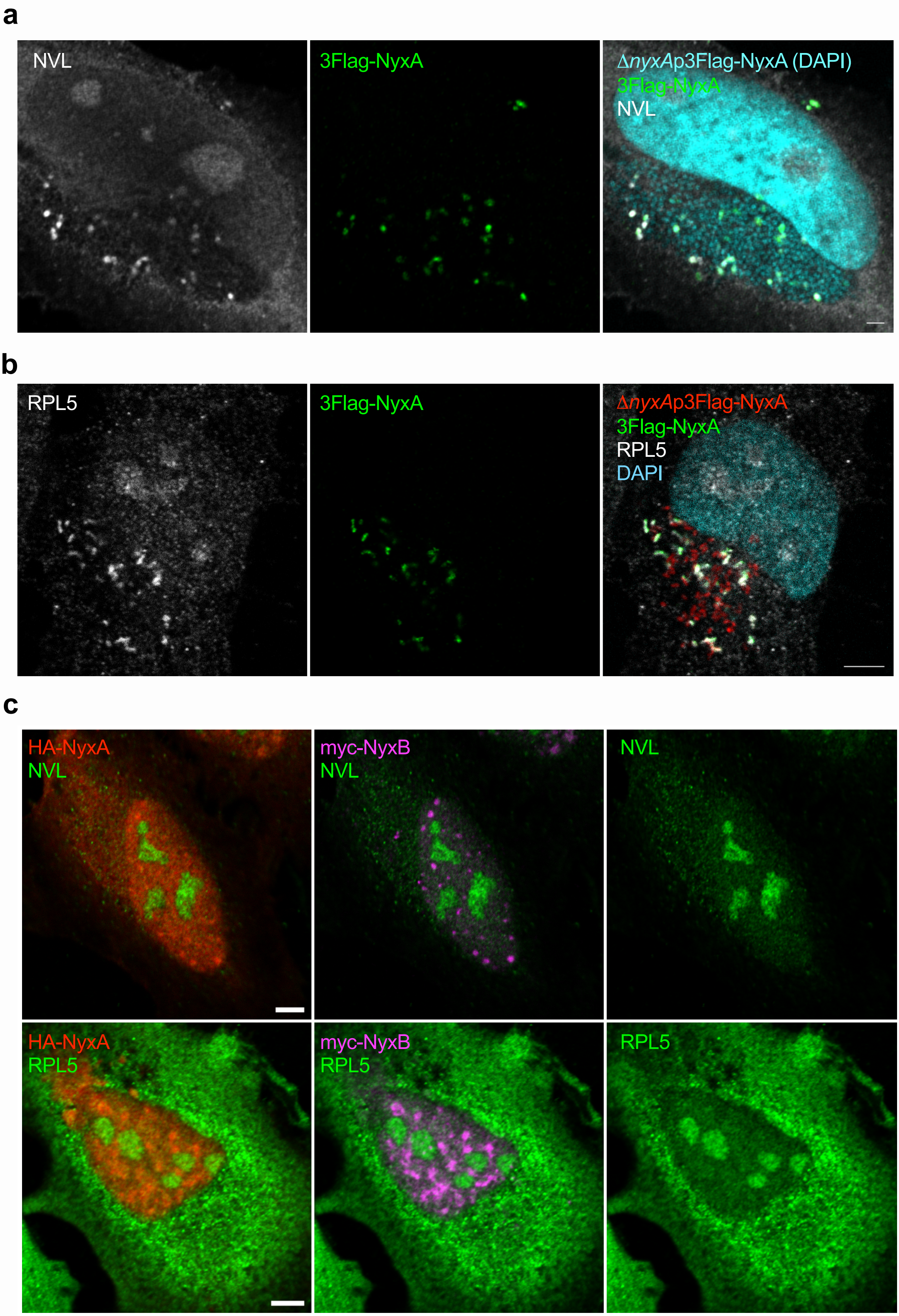

Supplementary Figure 9. *B. abortus* induces cytoplasmic accumulation of NVL and RPL5 that colocalises with translocated 3Flag-tagged NyxA. (a) HeLa cells were infected with *nyxA* expressing 3Flag-NyxA (green) for 48h and labelled for NVL (white) and DAPI (cyan). **(b)** HeLa cells were infected with *nyxA* expressing DSRed and 3Flag-NyxA (green) for 48h and labelled for RPL5 (white) and DAPI (cyan). **(c)** HeLa cells were transfected with both HA-NyxA and Myc-NyxB and labelled for NVL (top panel) or RPL5 (bottom panel). All scale bars correspond to 5µm.

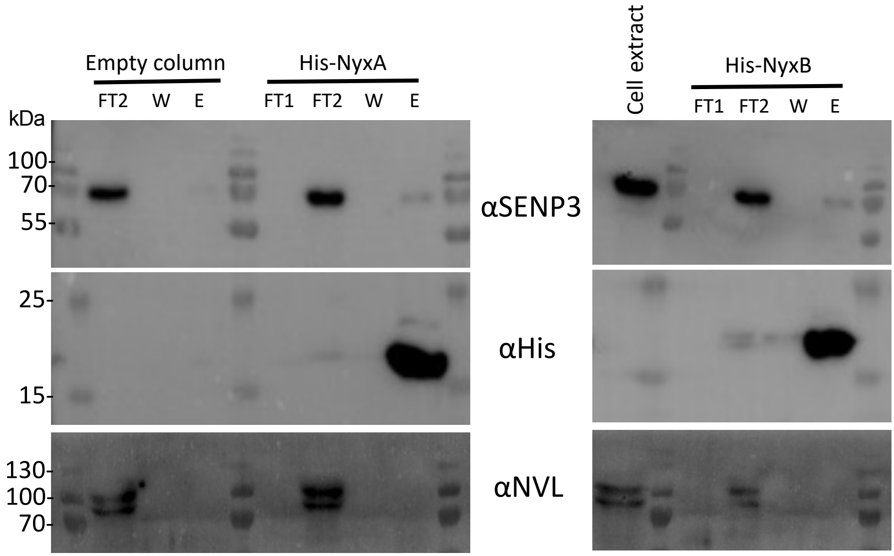

Supplementary Figure 10. NyxA and NyxB induce the formation of NVL-positive cytoplasmic structures without interacting with NVL. Pull-down assay with His-NyxA and His-NyxB immobilised on Ni NTA resins that were incubated with a HeLa cell extract. An empty column was used as a control for non-specific binding. Interactions with endogenous SENP3 and NVL were visualised by western blotting using the corresponding antibody and column binding with anti-His (lower blot). Non-bound fractions (F1 and F2), last wash (W) and elution (E) are shown for each sample and the molecular weights indicated (kDa). The cell extract input is also shown.

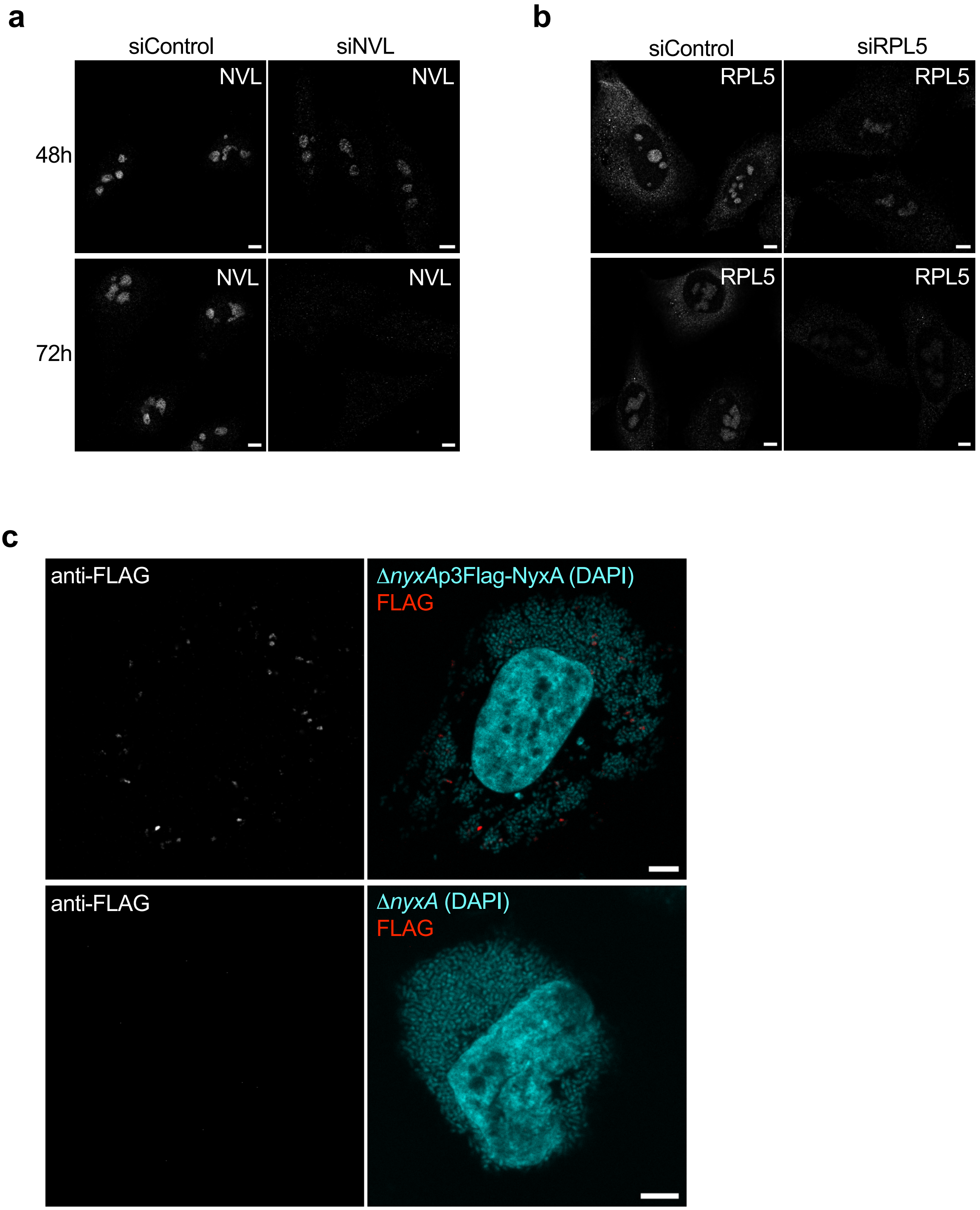

Supplementary Figure 11. Control experiments for the specificity of the antibodies used for the PLA. HeLa cells were treated with siRNA control or siNVL **(a)** or siRPL5 **(b)** for either 48h or 72h. Significant toxicity was observed, especially for 72h. Cells were then labelled with the anti-NVL or RPL5 antibody (from M. Nagahama) to verify their specificity. (**c**) The specificity of the FLAG antibody (SIGMA M2 clone) was confirmed in cells infected with *Brucella nyxA* strain not expressing 3FLAG-NyxA (bottom panel) in contrast to cells infected with *nyxA*p3FLAG-NyxA (top panel). Bacteria and nuclei were visualized with DAPI. All scale bars correspond to 5 μm.

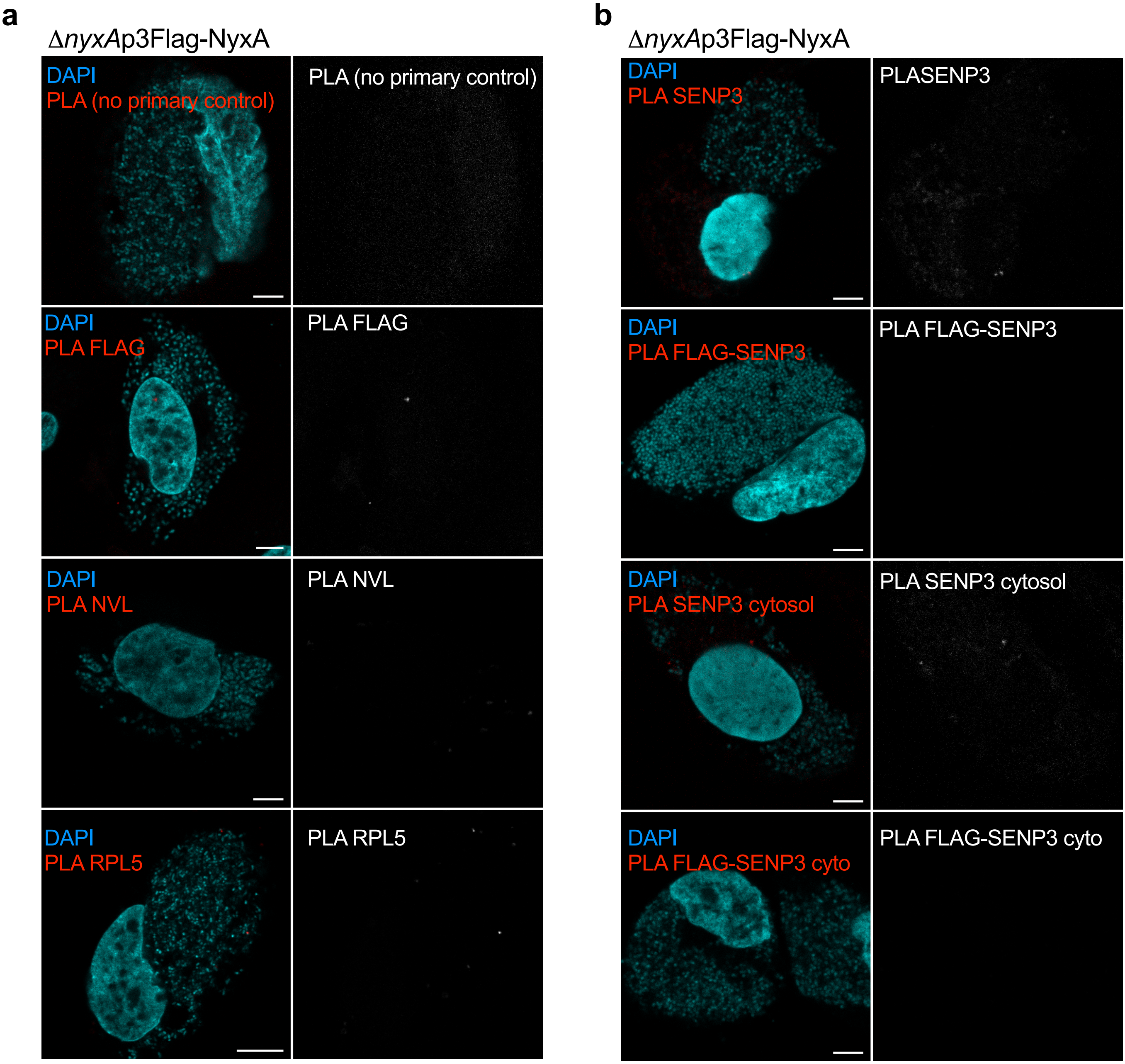

Supplementary Figure 12. Controls for PLA experiment. (a) Confocal images from control samples for the PLA experiments. HeLa cells infected with *nyxA*p3FLAG-NyxA were treated with the full protocol of the PLA without primary antibodies and each primary antibody alone (FLAG, NVL and RPL5). (**b**) The PLA experiments were also done for SENP3 alone and FLAG/SENP3 using either the standard protocol (top two panels) or a cytosolic labeling protocol (bottom two panels). All scale bars correspond to 5 μm.

cytosolic SENP3 detection protocol

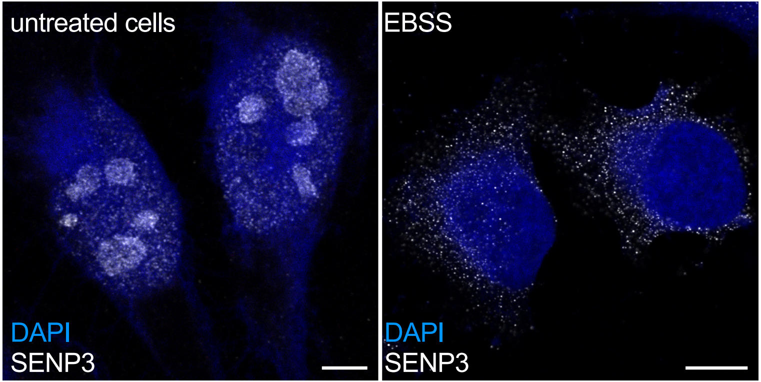

Supplementary Figure 13. Detection of cytosolic SENP3 during starvation conditions. Control HeLa cells were treated for EBSS for 24h before labeling. Scale bars correspond to 5 µm.

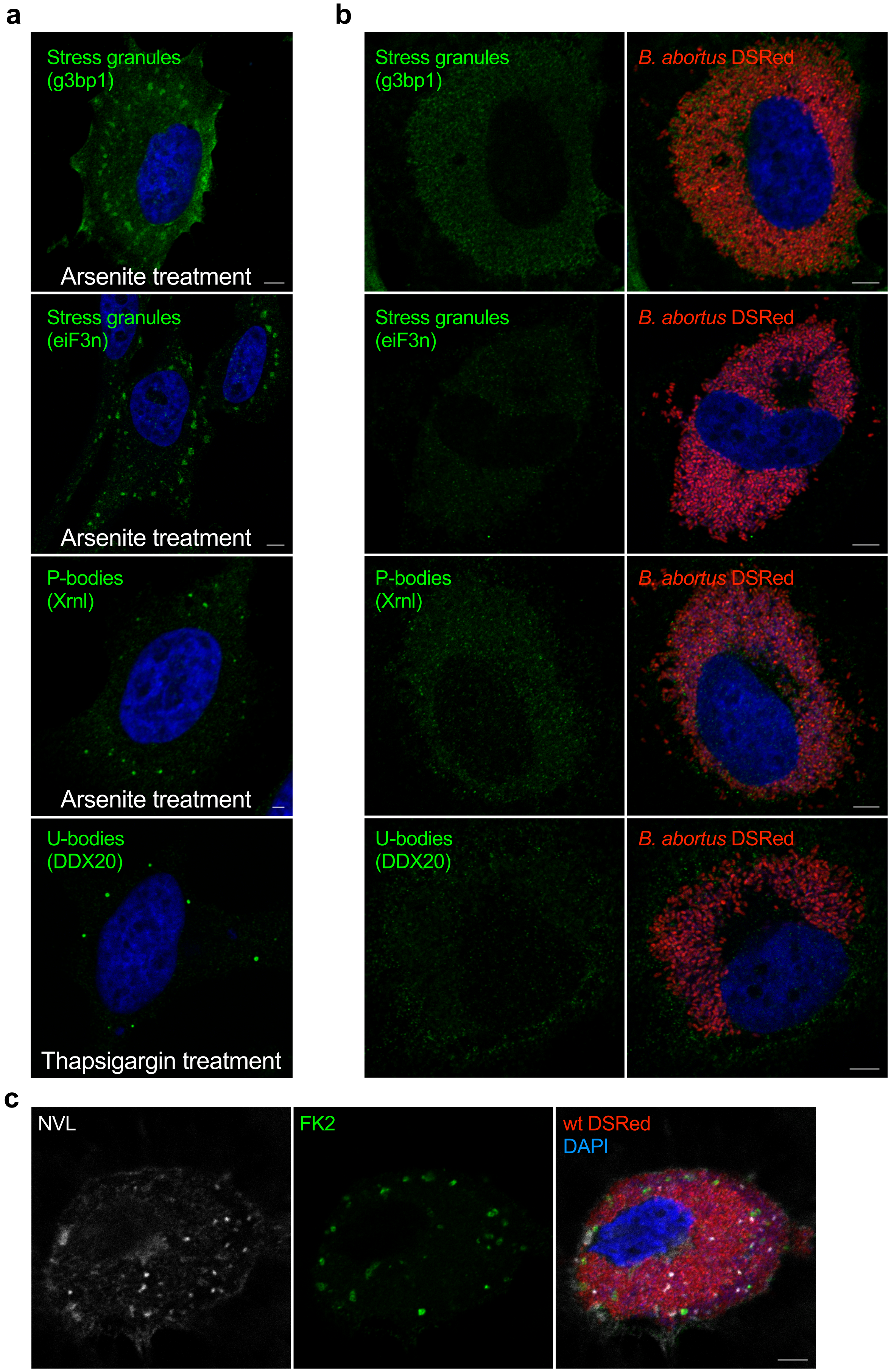

Supplementary Figure 14. *B. abortus* infection does not induce stress granules, P-bodies, or U-bodies. The cytoplasmic NVL structures are not FK2-positive. HeLa cells were either **(a)** treated with arsenite for 30 min or thapsigargin 4h as positive controls or **(b)** infected for 48h with wild-type DSRed-expressing *B. abortus* and labelled with anti-g3bp1 and eiF3n antibodies to visualise stress granules, anti-Xml antibody for P-bodies and anti-DDX20 antibody for U-bodies. NVL labelling was omitted from the figure for clarity. **(c)** Infected cells were also labelled with the FK2 antibody (green) that recognises mono- and poly-ubiquitinated proteins in addition to NVL (white). All scale bars correspond to 5 µm.

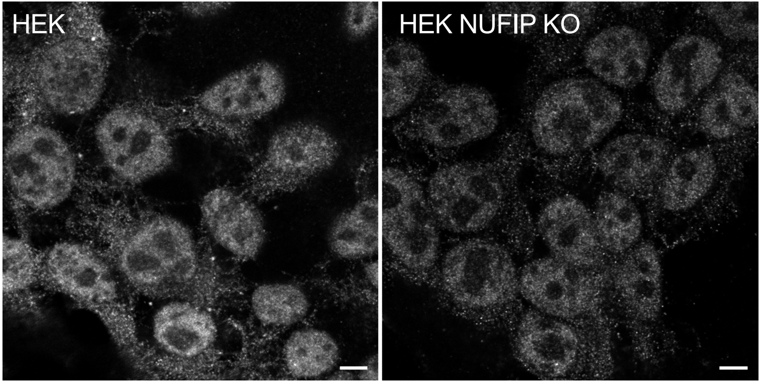

Supplementary Figure 15. Non-specific labeling of NUFIP antibody for immunofluorescence microscopy. Confocal image of HEK cells and HEK NUFIP CRISPR KO cells labelled for NUFIP (white). Scale bar corresponds to 5 µm.

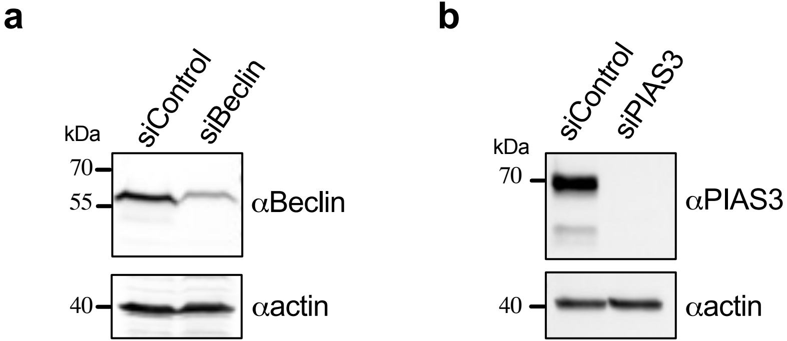

Supplementary Figure 16. siRNA depletion of Beclin1 and PIAS3. Western blots of cell lysates following 48h treatment with scrambled siRNA or **(a)** siBeclin1 or **(b)** PIAS3 and probed with anti-Beclin1 or PIAS3 antibodies, respectively. Control actin blots from the same membrane are shown underneath.

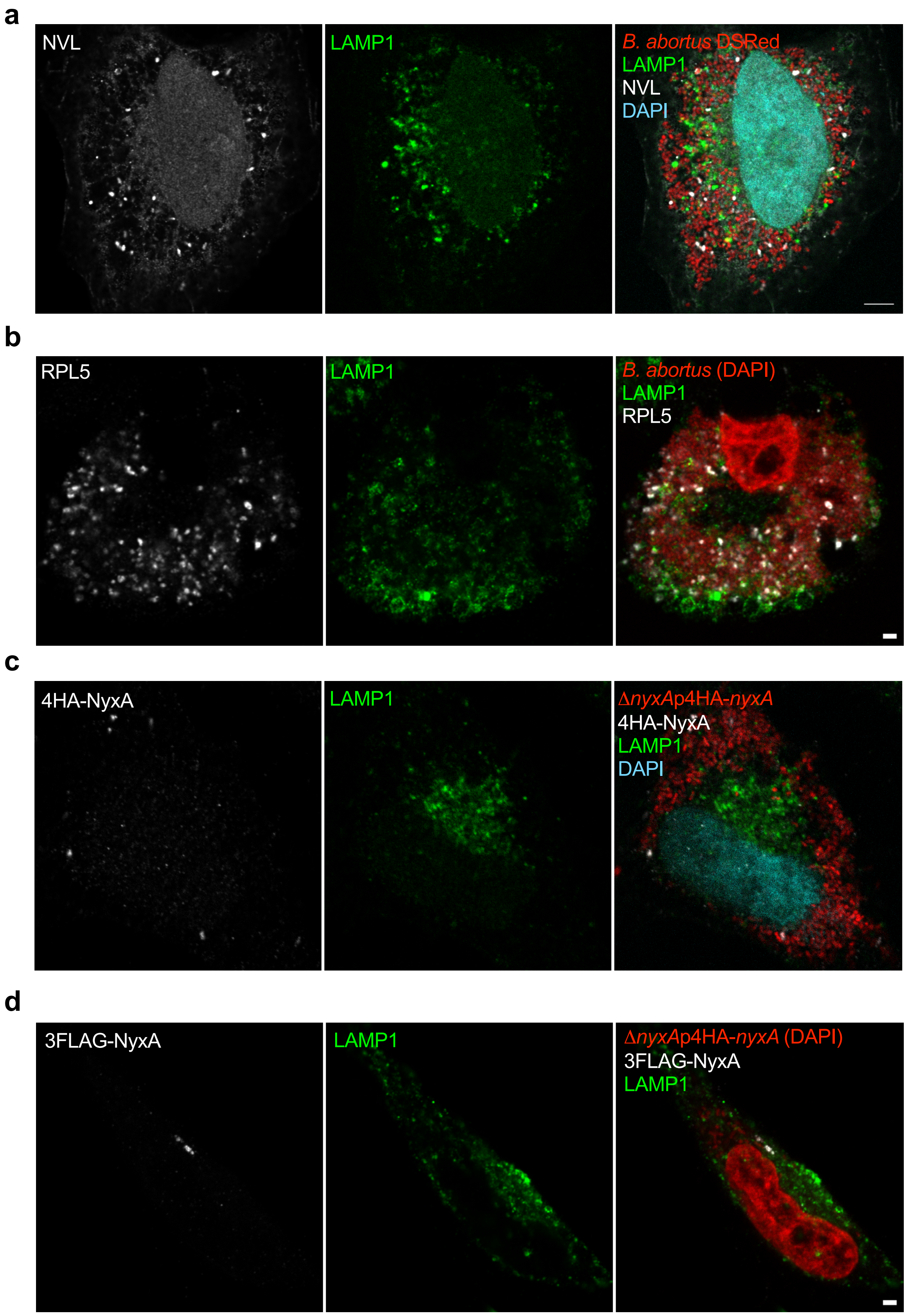

Supplementary Figure 17. Bif are negative for LAMP1. Representative confocal images of HeLa cells infected for 48h with **(a and b)** wild-type DSRed *B. abortus* or **(c)** *nyxA*p4HA-NyxA or **(D)** *nyxA*p3FLAG-NyxA**.** Cells were labelled for LAMP1 (green) with either NVL, RPL5, HA or FLAG, respectively. DAPI labeling was used to visualize nuclei and bacteria. Scale bar corresponds to 5 µm.

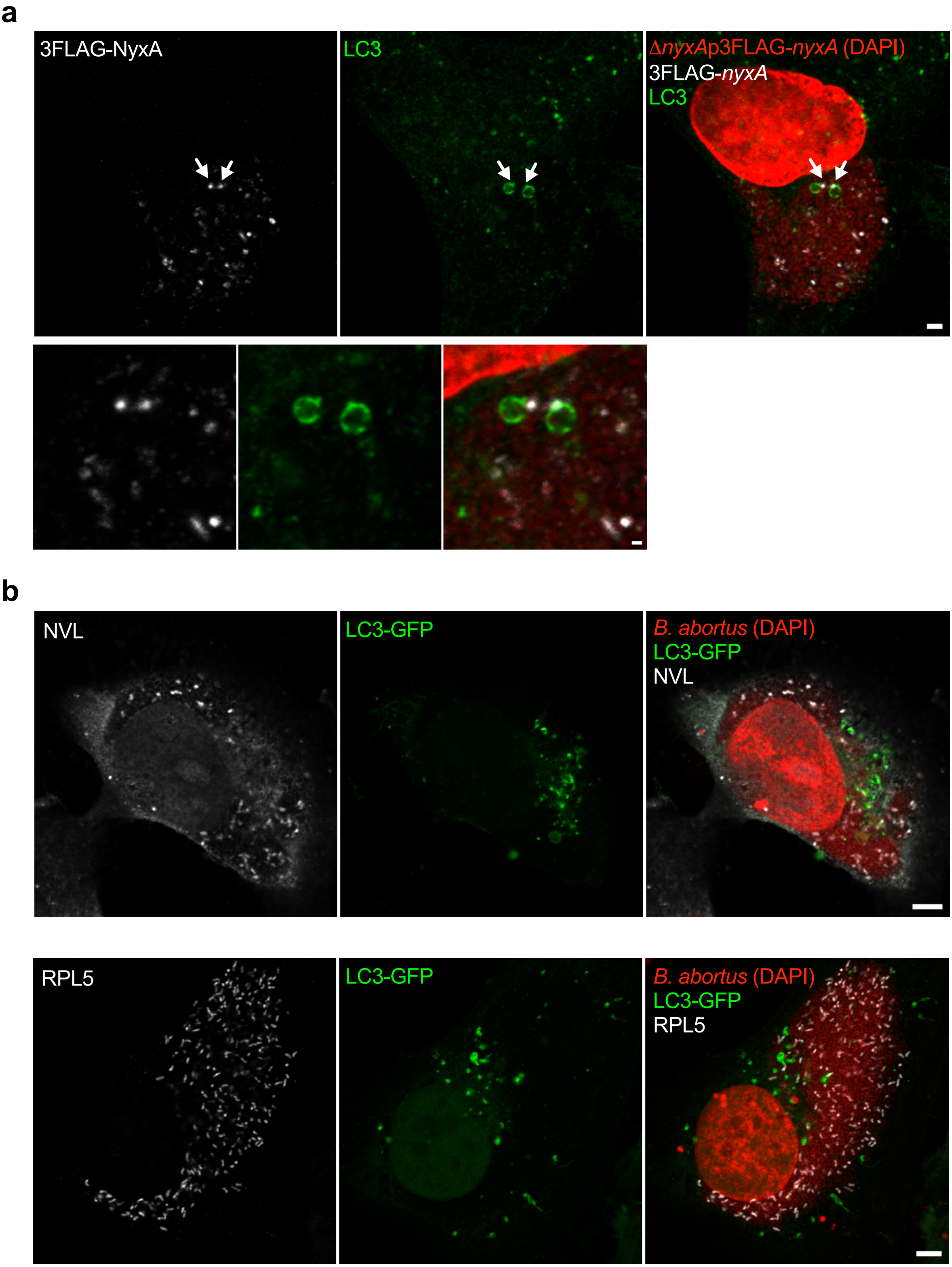

Supplementary Figure 18. Bif are negative for LC3. (a) Representative confocal images of HeLa cells infected for 48h with *nyxA*p3FLAG-NyxA and labelled for LC3 (green). Arrows indicate close contact between Bif and LC3-positive vacuoles, shown in inlets. **(b)** HeLa cells were infected for 24h with wild-type *B. abortus* and then transfected with LC3-GFP for another 24h. Cells were then fixed and labelled for NVL (white, top) or RPL5 (white, bottom). DAPI labeling was used to visualize nuclei and bacteria. Scale bar corresponds to 5 µm.

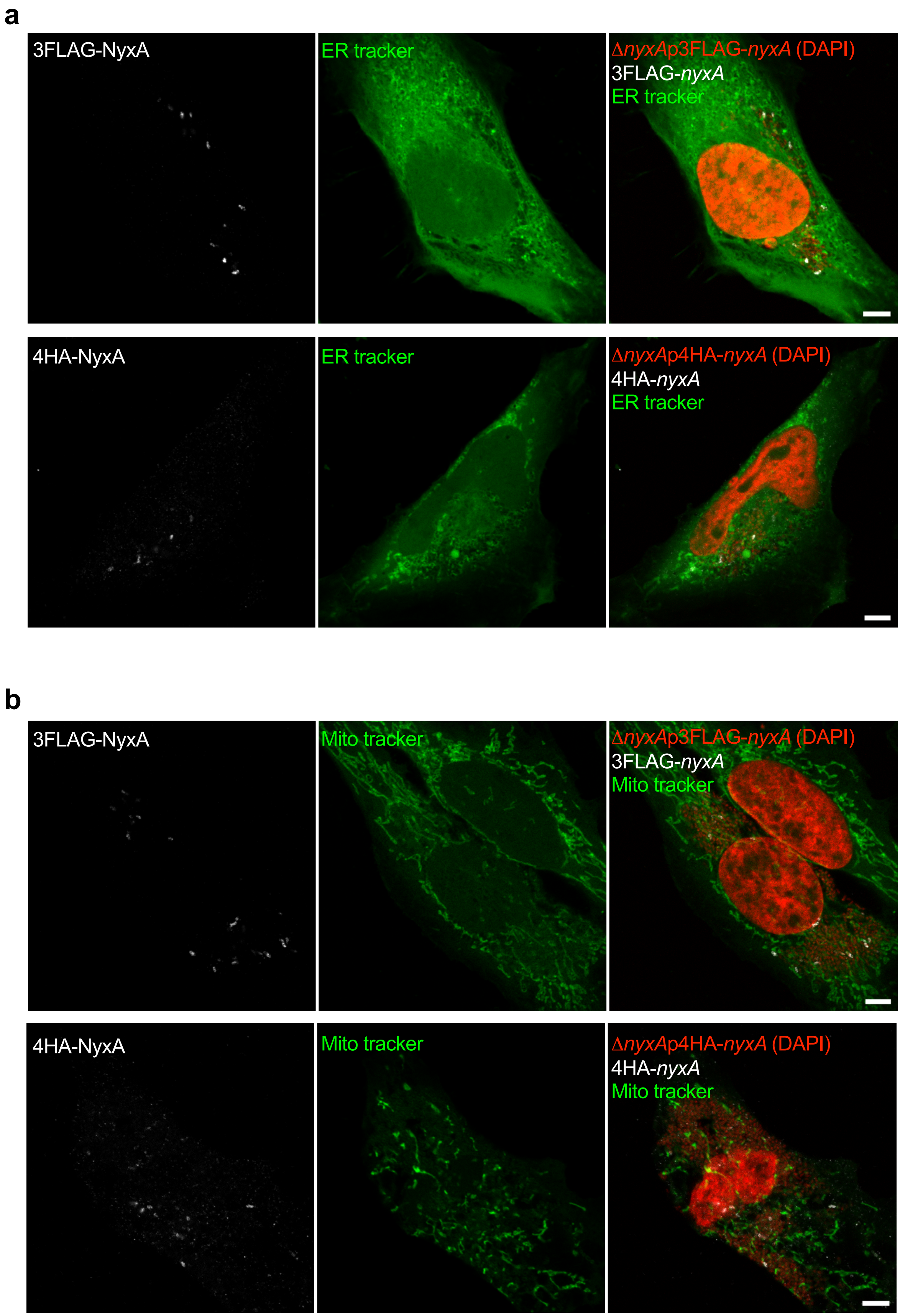

Supplementary Figure 19. Bif are negative for ER and Mitotrackers. Representative confocal images of HeLa cells infected for 48h with *nyxA*p3FLAG-NyxA or *nyxA*p4HA-NyxA and incubated with **(a)** ERtracker (green) or **(b)** Mitotracker (green). Cells were then fixed and labelled for either HA or FLAG (white). DAPI labeling was used to visualize nuclei and bacteria (red). Scale bar corresponds to 5 µm.

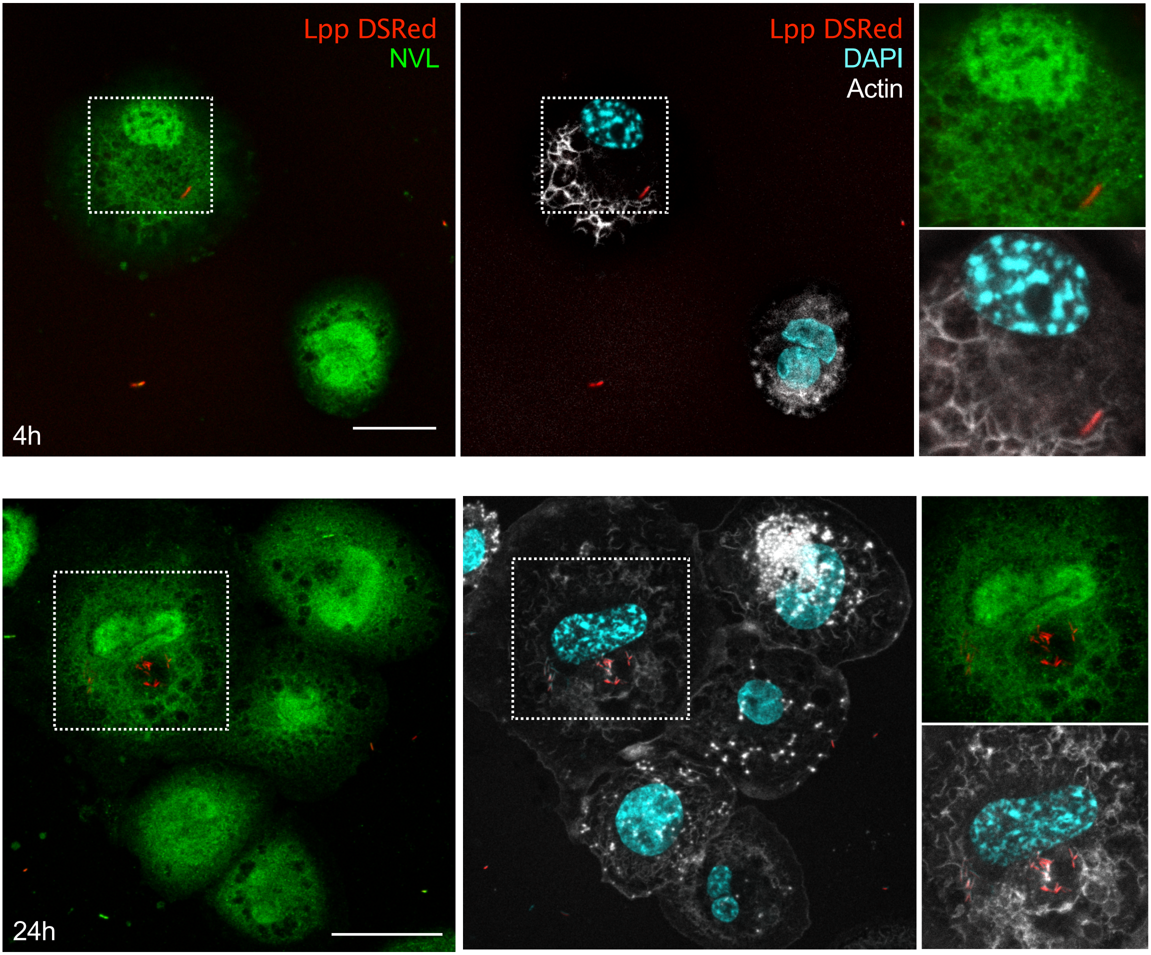

Supplementary Figure 20. *Legionella* infection does not induce NVL cytoplasmic accumulation. Immunofluorescence analysis of THP-1 cells infected 4 and 24 hours with wild type *L. pneumophila* strain Paris, carrying a DsRed expressing plasmid. Cells were stained with an anti-NVL antibody (green) and analysed by confocal microscopy. DAPI, light blue; Phalloidin, grey. Scale bars correspond to 10 µm.
